# The lung cancer pathway: seven years of evaluation of multidisciplinary pre-operative management and workflow optimization

**DOI:** 10.3389/fsurg.2026.1885436

**Published:** 2026-08-20

**Authors:** Adriana Nocera, Leonardo Petracca-Ciavarella, Maria Letizia Vita, Maria Teresa Congedo, Chiara Scognamiglio, Claudia Leoni, Claudia Bellettati, Alessia Senatore, Antonio Giulio Napolitano, Dania Nachira, Filippo Lococo, Elisa Meacci, Stefano Margaritora

**Affiliations:** Thoracic Surgery, Fondazione Policlinico Universitario “A.Gemelli” IRCCS, Università Cattolica del Sacro Cuore, Rome, Italy

**Keywords:** clinical pathway, lung cancer, multidisciplinary team, preoperative assessment, thoracic surgery

## Abstract

**Background:**

Multidisciplinary management is essential in thoracic oncology to optimize diagnostic assessment, treatment planning, and patient selection for surgery. Structured clinical pathways may improve coordination among specialists and reduce delays in the management of patients with suspected or confirmed lung cancer.

**Methods:**

We retrospectively analyzed patients managed within a structured Lung Cancer Pathway (LCP) at Fondazione Policlinico Universitario A. Gemelli IRCCS between January 2018 and December 2024. All patients entering the LCP were included, regardless of final treatment allocation. The pathway involved multidisciplinary assessment including thoracic surgeons, pulmonologists, oncologists, radiologists, pathologists, nuclear medicine specialists, and interventional specialists. The primary endpoints were:

- time from LCP initiation to clinical record closure;
- time from clinical record closure to surgical intervention.

Workflow times were analyzed using median values and interquartile ranges (IQR). Differences between 2018 and 2024 were assessed using the Mann–Whitney U test, while temporal trends across the study period were evaluated using the Kruskal–Wallis test.

**Results:**

A total of 2,691 patients were included. During the study period, the annual number of patients increased progressively, reflecting the expansion of the pathway. The median duration of the LCP significantly decreased from 9 days (IQR 2–23) in 2018 to 1 day (IQR 0–8) in 2024 (*p* < 0.001). A significant temporal trend was observed across the entire study period (*p* < 0.001). The median time from LCP closure to surgery was 29 days (IQR 15–43) in 2018 and 35 days (IQR 23–43) in 2024 (*p* = 0.013). During multidisciplinary evaluation, 447 patients (16.6%) did not proceed to surgery because of advanced disease, comorbidities, functional limitations, or patient preference.

**Conclusions:**

A structured Lung Cancer Pathway was associated with improved efficiency of the diagnostic and decision-making process over a seven-year period. The LCP model allowed standardized evaluation of patients with suspected or confirmed lung cancer, including those ultimately considered unsuitable for surgery. Although the retrospective design does not allow causal inference, these findings support the potential value of structured multidisciplinary pathways in optimizing workflow organization and clinical decision-making in thoracic oncology.

## 1 Introduction

Preoperative management of patients undergoing pulmonary resection is a critical determinant of surgical outcomes and postoperative recovery (1). Over recent years, thoracic surgery has progressively integrated multidisciplinary approaches aimed at optimizing the preoperative condition of lung cancer patients, especially those with high clinical complexity. The need for accurate pre-surgical assessment and timely risk stratification has led to the development of structured clinical pathways (CPs), which aim to standardize care, reduce variability, and improve both patient outcomes and healthcare efficiency.

Among these models, the “Lung Cancer Pathway” (LCP) at the Fondazione Policlinico Universitario A. Gemelli in Rome stands out as a comprehensive and effective approach to preoperative assessment. The LCP offers streamlined access to hematological and functional assessments (including ECG, spirometry, blood gas analysis), as well as multidisciplinary evaluations involving pulmonologists, cardiologists, and anesthesiologists—all conducted within a Day-Hospital setting.

This pathway enables a complete risk profile to be established, supporting timely decision-making and enhancing readiness for surgery.

Recent literature supports the relevance of such structured, preoperative programs. For example, prehabilitation—including pulmonary rehabilitation—has been shown to improve operability in patients previously considered unfit for resection (2). Similarly, virtual preoperative planning and enhanced coordination between specialties have been associated with a reduction in intraoperative complications and improved surgical precision in minimally invasive thoracic procedures (3).

Multidisciplinary preoperative assessment has been extensively discussed in European guidelines as essential for accurate surgical candidacy determination, particularly emphasizing cardiopulmonary function evaluation (4).

Other feasibility studies have demonstrated that preoperative optimization clinics (pre-hab services) are not only viable but also effective in enhancing the quality of care for lung cancer patients (5).

In this context, the present study aims to evaluate the effectiveness of the Lung Cancer Pathway (LCP) implemented at the Fondazione Policlinico Universitario Agostino Gemelli in optimizing the pre-hospitalization and diagnostic workflow for surgical patients with lung neoplasms. Special emphasis is placed on the role of a multidisciplinary framework in improving access to care, reducing delays, and ensuring that even high-complexity patients are thoroughly assessed with a minimal number of hospital visits, thereby enhancing efficiency without compromising clinical thoroughness.

## 2 Materials and methods

### 2.1 Ethical statement

This is a retrospective, single-center observational study conducted at Fondazione Policlinico Universitario “A. Gemelli” IRCCS—Università Cattolica del Sacro Cuore (Rome, Italy).

The study was performed as a retrospective service evaluation based on routinely collected clinical data from the Lung Cancer Pathway (LCP). The Institutional Review Board (IRB) of the Catholic University of the Sacred Heart was informed about the study design. Since the analysis involved only anonymized retrospective data, did not modify patient management, and was conducted within the framework of an institutional clinical audit, formal IRB approval was not required.

Clinical and demographic variables were retrospectively extracted by authorized investigators from the institutional electronic medical records of patients included in the LCP. The dataset was anonymized before statistical analysis in accordance with institutional policy.

All patients provided written informed consent for diagnostic and therapeutic procedures, including authorization for the anonymized use of their clinical information according to institutional policy.

According to institutional policy, no formal IRB protocol number was assigned for this retrospective service evaluation based on anonymized routinely collected clinical data.

### 2.2 Inclusion and exclusion criteria

Patients were retrospectively identified from the LCP database between January 1, 2018 and December 31, 2024. All patients entering the LCP were included, regardless of the final treatment allocation. Inclusion criteria consisted of patients with either histologically confirmed primary lung cancer or radiologically highly suspicious pulmonary lesions requiring multidisciplinary evaluation and considered potential candidates for surgical treatment based on initial clinical assessment. Patients were not excluded based on disease stage or final therapeutic decision, as one of the objectives of the LCP is to allow multidisciplinary assessment and identification of patients who are not suitable for surgical treatment because of advanced disease, comorbidities, functional impairment, or patient preference.

### 2.3 Preoperative evaluation protocol

The pre-hospitalization pathway of the LCP program was managed in a Day Hospital setting. Each patient underwent a structured diagnostic workup that included:

- Complete blood count and serum biochemistry panel
- Resting 12-lead electrocardiogram (ECG)
- Pulmonary function tests (PFTs), including spirometry and arterial blood gas analysis
- Specialist evaluations: pulmonology, cardiology (in patients with known or suspected cardiovascular disease), and anesthesiology. Cardiology consultation was requested in patients with known or suspected cardiovascular disease or when clinically indicated during the preoperative assessment.

The Day Hospital model does not imply that all investigations are completed within a single day; rather, it refers to a structured outpatient organizational pathway allowing patients to undergo standardized diagnostic evaluations and specialist consultations without conventional hospitalization.

A comprehensive radiological staging was also performed using contrast-enhanced total body computed tomography (CT) and positron emission tomography (PET). When feasible, histological characterization of the lesion was obtained through bronchoscopy or CT-guided biopsy.

**Figure 1:**
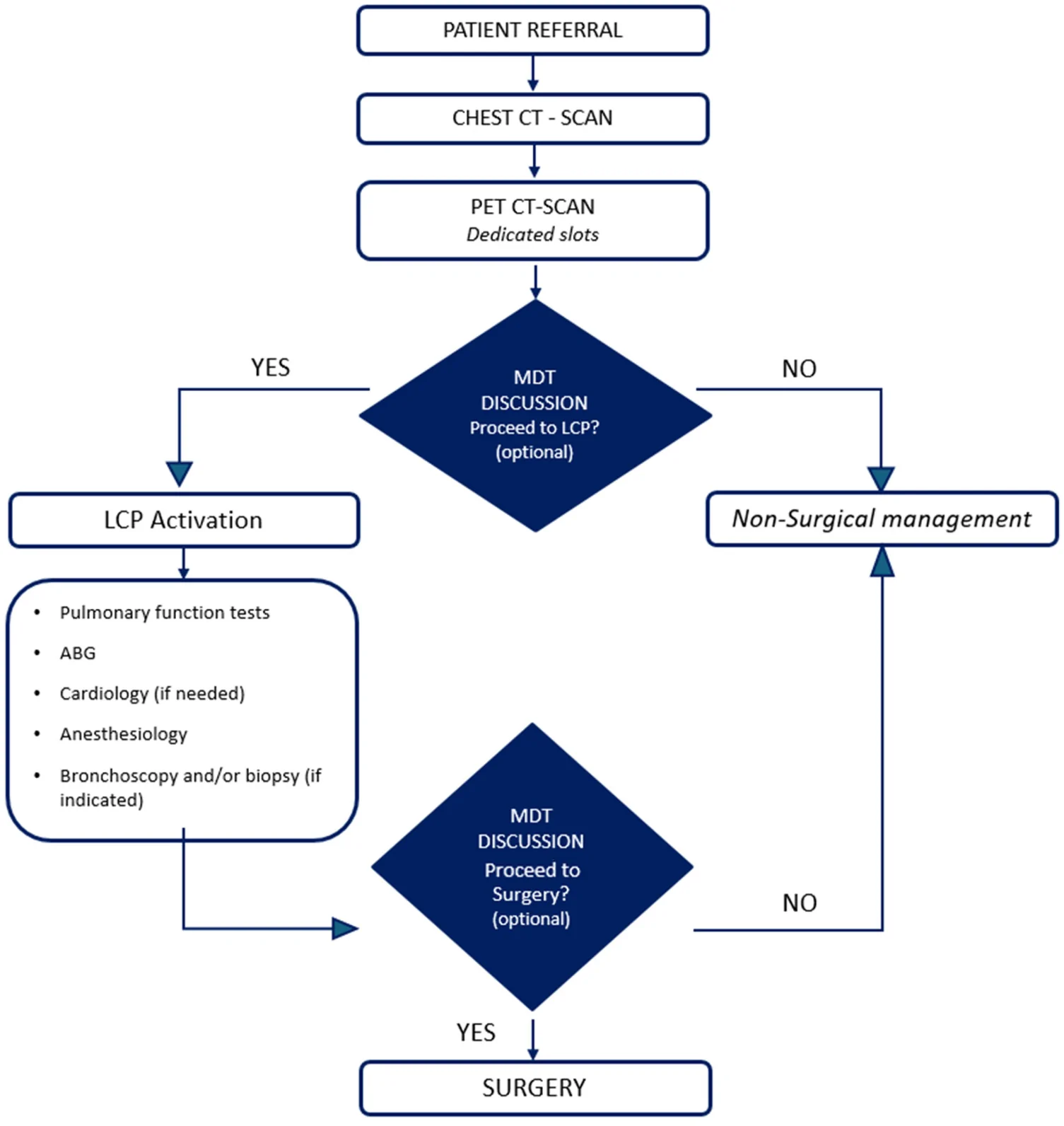
Schematic representation of the lung cancer pathway (LCP). Patients undergo initial radiological assessment before entering the LCP. Multidisciplinary Team (MDT) discussions are performed whenever clinically indicated during the diagnostic pathway to guide treatment allocation.

PET-CT represented a mandatory step before activation of the LCP and was performed through dedicated weekly institutional slots reserved for patients entering the pathway. Whenever technically feasible and clinically indicated, tissue diagnosis by bronchoscopy or CT-guided biopsy was obtained after PET-CT and before definitive treatment planning. Additional staging procedures, including endobronchial ultrasound-guided transbronchial needle aspiration (EBUS-TBNA) or other investigations, were performed whenever clinically indicated during the diagnostic pathway (Figure 1).

In high-complexity cases, additional second- and third-level diagnostic procedures were included, such as echocardiography, cardiopulmonary exercise testing (CPET), and coronary CT angiography. Anesthetic risk was stratified according to the American Society of Anesthesiologists (ASA) Physical Status Classification System (Table 1).

**Table 1:** Clinical, functional, and imaging tests required as part of the preoperative evaluation protocol for surgical candidates.

LCP Phase	Evaluation	Description
*Diagnostic-Therapeutic Workup*	Thoracic Surgeon	- Remote medical history - Total body CT with contrast - PET with 18F-FDG
	Multidisciplinary Discussion (MDT)	
*Pre-operative Workup: Step I*	Pulmonologist	- Spirometry - DLCO - Blood gas analysis
	Cardiologist consultation when clinically indicated	- ECG - Echocardiogram
	Anesthesiologist	- Blood chemistry tests (coagulation, CBC, electrolytes)
*Pre-operative Workup: Step II*	Pulmonologist	- 6-minute walking test - Perfusion scintigraphy
	Cardiologist	- ECG-stress test - Pharmacological Echo-stress
	Specialty Examinations by Anesthesiologist	- For example, fibrolaryngoscopy for intubation evaluation
*Pre-operative Workup: Step III*	Cardiologist	- Myocardial scintigraphy - Coronary angiography
	Pulmonologist	- Cardiopulmonary stress test
	Specialty Evaluations if Needed or Multidisciplinary Discussion (MDT)	

Fitness for surgery was assessed according to current international recommendations adopted in our institution, integrating pulmonary function tests, arterial blood gas analysis, cardiological evaluation when indicated, and cardiopulmonary exercise testing in selected patients.

### 2.4 Multidisciplinary management and tumor board

The Multidisciplinary Team (MDT—Tumor Board) represented the core decision-making component of the LCP.

Entry into the LCP did not necessarily require a previous MDT discussion. Patients were referred to the LCP after completion of the initial radiological assessment, including chest CT and PET-CT. MDT discussion was performed whenever clinically indicated, either before or during the LCP, according to case complexity, diagnostic findings, and therapeutic decision-making needs.

The MDT included thoracic surgeons, medical oncologists, pulmonologists, radiologists, pathologists, interventional pulmonologists, interventional radiologists, and nuclear medicine specialists.

Rehabilitation specialists, nutritionists, and other allied healthcare professionals were not permanent members of the MDT during the study period. However, additional specialist consultations were requested on an individual basis when clinically required, including during hospitalization or subsequent clinical management.

### 2.5 Smoking cessation management

Active smokers received counseling regarding smoking cessation during the preoperative evaluation process. Although a standardized smoking cessation program was not formally integrated into the LCP, patients expressing the desire or need for additional support could be referred to a dedicated addiction specialist with expertise in tobacco dependence management.

### 2.6 Preoperative pulmonary rehabilitation

A standardized preoperative pulmonary rehabilitation program was not routinely included within the LCP during the study period. Patients with specific functional limitations were managed individually according to clinical indication and could undergo targeted respiratory optimization when considered appropriate.

### 2.7 Statistical analysis

Demographic and clinical variables were retrospectively collected from electronic medical records.

Continuous variables related to workflow duration were assessed for distribution characteristics and were considered non-normally distributed. Therefore, time intervals are reported as median and interquartile range (IQR).

The primary workflow endpoints were:

- time from LCP initiation to clinical record closure;
- time from clinical record closure to surgical intervention.

Comparisons between independent yearly cohorts were performed using the Mann–Whitney U test for two-group comparisons (2018 vs. 2024). Temporal trends across the entire study period (2018–2024) were evaluated using the Kruskal–Wallis test.

Categorical variables were expressed as absolute numbers and percentages.

A two-sided *p*-value <0.05 was considered statistically significant.

### 2.8 Primary and secondary objectives

Primary Objectives:

To assess the efficiency of the Lung Cancer Pathway (LCP) in the preoperative management of patients with pulmonary neoplasms who are candidates for surgery, by measuring:

- the median time (days) required to complete the LCP, from the first evaluation to clinical record closure;
- the median time (days) between LCP closure and surgical intervention among patients considered suitable for surgery.

Secondary Objectives:

To evaluate clinical complexity and appropriateness of the pathway, by analyzing:

- The number and annual percentage of patients requiring at least one additional specialist consultation beyond the standard pathway (e.g., cardiology, infectious diseases, endocrinology).
- the proportion of patients not proceeding to surgery and the reasons for non-operative management, including advanced disease, comorbidities, functional limitations, or patient refusal;
- annual changes in pathway volume and complexity.

## 3 Results

### 3.1 Study population

Between January 1, 2018 and December 31, 2024, a total of 2,691 patients were enrolled in the Lung Cancer Pathway (LCP) and were included in the analysis.

The annual number of patients increased progressively during the study period, from 290 patients in 2018 to 508 patients in 2024, reflecting the progressive expansion of the pathway.

Among the overall population, 1,469 patients (54.6%) were male and 1,222 (45.4%) were female.

Regarding smoking habits, 52.2% of patients were current or former smokers, while 48,8% had never smoked.

Anesthesiologic risk was stratified using the ASA Physical Status Classification: 0,6% were classified as ASA I, 54,9% as ASA II, 44,25% as ASA III, and 0,08% as ASA IV (Table 2).

**Table 2:** Baseline characteristics of patients enrolled in the LCP.

Variables	*n* (%)
Gender	
M	1,469 (54,6%)
F	1,222 (45,4%)
Smoking status	
Smoker or Ex	1,379 (51,2%)
Non-smoker	1,312 (48,8%)
ASA score (surgical candidates *n* = 2,244)	
ASA I	15 (0,6%)
ASA II	1,234 (54,9%)
ASA III	993 (44,25%)
ASA IV	2 (0,08%)

These data indicate that the majority of patients undergoing evaluation presented relevant comorbidity burden, with almost half classified as ASA III.

### 3.2 Primary objectives

#### 3.2.1 Average duration of the LCP pathway and time to surgical intervention

The distribution of workflow-related time intervals was non-normal; therefore, results are presented as median and interquartile range (IQR). The median duration of the LCP progressively decreased during the study period. In 2018, the median time from pathway initiation to clinical record closure was: 9 days (IQR: 2–23) while in 2024 it decreased to 1 day (IQR: 0–8).

The difference between 2018 and 2024 was statistically significant *p* < 0.001. The overall temporal trend from 2018 to 2024 was also statistically significant *p* < 0.001.

These findings demonstrate a progressive optimization of the diagnostic and organizational workflow over time. The median interval between clinical record closure and surgical intervention showed a different pattern. In 2018, 29 days (IQR: 15–43) while in 2024 35 days (IQR: 23–43). Although no progressive reduction was observed in this interval, yearly variation remained significant (*p* < 0.001).

The interval between LCP closure and surgery should be interpreted separately from diagnostic workflow efficiency, as it depends on multiple organizational and clinical factors, including operating room scheduling, patient optimization, and surgical prioritization.

The comparison between 2018 and 2024 showed *p* = 0.013.

This finding suggests that while the preoperative assessment process became increasingly efficient, the interval between multidisciplinary decision and surgery may depend on additional organizational factors, including operating room availability, clinical prioritization, and complexity of surgical planning (Table 3).

**Table 3:** Workflow timing of the lung cancer pathway between 2018 and 2024. Data are expressed as median (interquartile range). A recorded duration of 0 days indicates completion of the corresponding LCP step within the same calendar day and should be interpreted as completion within one day.

Year	LCP initiation to clinical record closure, days	Clinical record closure to surgery, days
*2018*	9 (2–23)	29 (15–43)
*2019*	6 (1–17.5)	26 (15–43)
*2020*	2 (0–11)	14 (8–25)
*2021*	1 (0–8.25)	20 (14–31)
*2022*	1 (0–7)	22 (14–36)
*2023*	1 (0–6)	24 (16–36)
*2024*	1 (0–8)	35 (23–43)

### 3.3 Secondary objectives

#### 3.3.1 Clinical complexity and additional assessments

All patients underwent the standard multidisciplinary preoperative assessment.

All patients enrolled in the LCP underwent standard preoperative evaluations, including pulmonary, anesthesiologic, and thoracic surgical assessments. During the study period, 640 patients (23.8%) required at least one additional specialist consultation beyond the core evaluations. The most frequently requested additional assessments were cardiology in 531 patients, hematology in 21 patients, and infectious diseases in 15 patients.

Other specialist evaluations were performed according to individual clinical needs and were not considered permanent components of the MTB.

**Figure 2:**
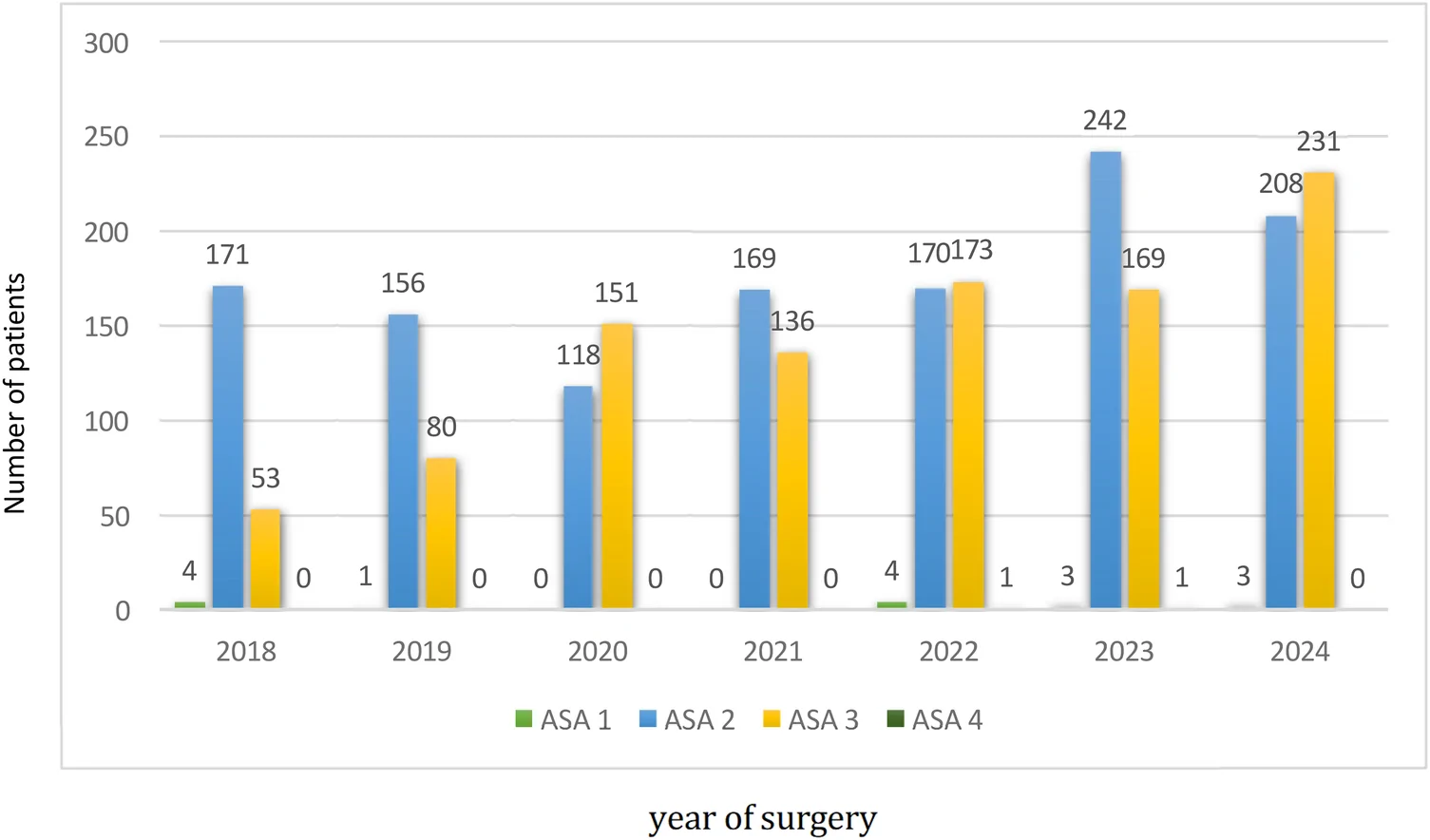
Annual distribution of ASA score (1–4) among patients who underwent surgery within the LCP between 2018 and 2024.

In 2024, ASA 3 patients represented the relative majority of operated cases, accounting for 52.3% of the total. This marks a significant increase compared to previous years, when the proportion of ASA 3 patients was lower. This suggests a possible increase in patient complexity over time, as inferred by ASA classification, although this remains a proxy measure and should be interpreted with caution (Figure 2).

#### 3.3.2 Annual enrollment trend in the LCP and surgical ineligibility

In total, the LCP saw an increase from 290 patients in 2018 to 508 patients in 2024.

Overall, 447 patients (16.6%) were deemed not eligible for surgery.

The main reasons for exclusion were:

- patient refusal: 232 patients (51.9% of non-operated patients)
- significant comorbidities or functional limitations: 163 patients (36.5%)
- advanced disease stage identified during diagnostic work-up: 46 patients 10.3%)

Importantly, these patients were included in the LCP analysis, as multidisciplinary evaluation was performed regardless of final treatment allocation.

## 4 Discussion

Our study evaluates a seven-year experience with a structured Lung Cancer Pathway (LCP) designed to optimize preoperative assessment and multidisciplinary decision-making in patients with suspected or confirmed pulmonary neoplasms.

The main finding of this analysis is that the implementation of a structured multidisciplinary pathway was associated with a progressive improvement in the efficiency of the preoperative workflow, particularly regarding the time required to complete clinical evaluation and reach treatment planning.

The progressive reduction in LCP duration observed between 2018 and 2024 suggests that the pathway became increasingly standardized over time, despite the progressive increase in annual patient volume and the management of clinically complex cases.

Previous studies have demonstrated the importance of structured clinical pathways and multidisciplinary models in thoracic oncology, particularly in reducing variability of care, improving coordination between specialists, and facilitating timely treatment decisions (6–8).

In our experience, the LCP model allowed patients to undergo a coordinated diagnostic and functional assessment within a dedicated Day Hospital framework. This organizational model does not necessarily imply completion of all investigations within a single day, but rather the availability of a structured pathway that minimizes unnecessary hospital admissions and optimizes scheduling of diagnostic procedures.

The temporary increase in pathway duration observed in 2022 may be related to organizational consequences of the COVID-19 pandemic, which affected outpatient activity and healthcare resource availability. However, the overall trend remained consistent with progressive workflow optimization.

Previous experiences have highlighted that centralized multidisciplinary approaches and structured preoperative pathways may improve resource utilization and reduce delays in patients undergoing pulmonary resection (9–11). Our findings are consistent with these observations, showing that pathway organization may facilitate rapid identification of surgical candidates while maintaining comprehensive clinical evaluation.

A crucial component in the pre-hospitalization of these patients is the pulmonary assessment, which plays a key role in determining the patient's lung reserve and predicting the risk of postoperative complications (12). The use of spirometry and arterial blood gas analysis (13, 14) allowed for the early identification of patients with reduced pulmonary function, enabling timely management of respiratory conditions (15, 16).

The present study does not evaluate postoperative outcomes directly; therefore, the role of the LCP should be interpreted as an organizational model supporting appropriate patient selection and multidisciplinary decision-making rather than as a direct predictor of surgical results.

Cardiological assessment is equally crucial in the preoperative evaluation, particularly in thoracic surgery, where cardiovascular comorbidities are frequently encountered. The high number of cardiology consultations requested (531 patients, accounting for 19.7% of the cohort) highlights the increasing need for a multidisciplinary approach in the management of patients undergoing thoracic surgery. In our clinical pathway, cardiological risk stratification based on tests such as electrocardiograms (ECG), echocardiograms, and ejection fraction measurements enabled the timely identification of patients at elevated cardiovascular risk (17).

This approach is consistent with findings in the literature, where highlight that preoperative cardiological evaluation significantly reduces the risk of perioperative cardiovascular events, such as heart attacks or heart failure (18).

In our case, patients with heart failure or pre-existing coronary artery disease were adequately managed with preoperative optimization of medications and monitoring, minimizing complications during and after the procedure. As emphasized Salvatici et al. (19) careful cardiovascular evaluation allows for timely interventions that improve surgical outcomes.

Cardiology was not considered a permanent component of the MDT; rather, cardiological evaluation was requested according to individual patient characteristics and clinical indications.

Anesthesiological evaluation plays a fundamental role in assessing operative risk and planning perioperative management. However, ASA classification should be considered a surrogate marker of clinical complexity and should not be interpreted as an independent measure of surgical outcome. The integration between thoracic surgeons and anesthesiologists allowed individualized evaluation and risk adaptation, consistent with the principles of multidisciplinary perioperative optimization (20–22).

All pulmonary resections, whether anatomical or non-anatomical, performed via a minimally invasive approach (Uniportal VATS) or open surgery, were conducted under general anesthesia with single-lung ventilation (20).

An important aspect emerging from our experience is the role of timely communication between the surgical and anesthesiology teams, which enables the development of a personalized perioperative management plan.

This integrated approach is supported by Shargall et al., who emphasized the value of multidisciplinary coordination in minimizing anesthetic risks, particularly in high-complexity patients (22).

The role of the multidisciplinary team (MDT) in determining the appropriate treatment for patients who are not suitable for surgery is another key element of our experience. As highlighted by Brunelli et al. (5), discussing complex cases in a multidisciplinary setting allows for the exploration of various therapeutic options such as chemotherapy, radiotherapy, or immunotherapyand helps optimize treatment for each patient. In our study, patients deemed unsuitable for surgery were promptly referred for personalized oncological treatments, thus avoiding delays in the initiation of therapy (23).

Importantly, these patients were not excluded from the pathway, as the objective of the LCP is not only to identify surgical candidates but also to ensure appropriate management of patients requiring alternative therapeutic strategies.

Furthermore, the discussion of strategies for accurate histological typing of tumors, particularly in patients who are not candidates for surgical resection, is essential to ensure the most tailored and effective treatment approach (10–24).

The ability of a multidisciplinary tumor board to manage and adapt therapeutic pathways is essential, and our findings support the role of the MDT in facilitating individualized treatment planning and timely referral to the most appropriate therapeutic strategy.

Smoking cessation represents an important aspect of perioperative optimization. Although a standardized smoking cessation program was not incorporated into the LCP during the study period, active smokers received counseling and patients requiring additional support could be referred to specialists with expertise in tobacco dependence.

Similarly, a standardized preoperative pulmonary rehabilitation program was not routinely included in the pathway. Respiratory optimization strategies were considered on an individual basis according to patient functional status and clinical indication.

### 4.1 Study strengths, limitations, and future directions

This study presents several strengths. First, it is based on a large cohort (*n* = 2,691) managed over seven years, reflecting real-world experience from a high-volume tertiary center. The standardized and well-established nature of the LCP allowed for the analysis of consistent and comparable data. The integration of multidisciplinary teams within a Day-Hospital setting offers an efficient and scalable organizational model. Moreover, the ability to manage high-complexity patients with a limited number of hospital visits confirms the pathway's operational efficiency without compromising clinical thoroughness.

Several limitations must be acknowledged. The retrospective single-center design limits generalizability. The absence of a comparator group represents a limitation of this study. Since the LCP was implemented as the standard organizational pathway for all patients evaluated in our institution, an internal untreated control group was not feasible.

Furthermore, workflow time variables were analyzed as organizational indicators and not as surrogate markers of improved survival, surgical outcomes, or patient-reported quality of life.

Future prospective studies including clinical outcomes, patient satisfaction, and economic evaluation will be necessary to further validate the impact of structured lung cancer pathways.

In the future, the integration of artificial intelligence and machine learning tools could further optimize clinical data collection and analysis, enhancing the personalization of the preoperative pathway and supporting faster, more accurate decision-making. Additionally, a cost-effectiveness analysis could strengthen the adoption of the model on a larger scale.

## 5 Conclusion

The Lung Cancer Pathway (LCP) implemented at Fondazione policlinico Universitario A. Gemelli IRCCS represents a structured multidisciplinary model for the evaluation and management of patients with suspected or confirmed pulmonary neoplasms.

Over the seven-year study period, the LCP was associated with a progressive reduction in the time required to complete multidisciplinary assessment and finalize diagnostic-therapeutic planning.

The integration of the thoracic surgeons, pulmonologists, oncologists, radiologists, pathologists, nuclear medicine specialists, and interventional specialists supported standardized evaluation and individualized decision-making.

The inclusion of the all patients entering the pathway, regardless of final treatment allocation, allowed the multidisciplinary team board to identify both surgical candidates and patients requiring alternative therapeutic strategies.

The Day Hospital organizational model facilitated coordinated assessment within a structured clinical pathway.

Although this retrospective study does not allow causal inference regarding the effectiveness of the LCP or its impact on clinical outcomes, the findings support the potential valuea of structured multidisciplinary pathways in improving workflow organization and standardizing care processes in thoracic oncology.

Future prospective and multicenter studies including clinical outcomes, quality-of-life measures, patient satisfactin, and cost-effectiveness analyses will be required to further evaluate the broader impact of this model.

## Data Availability

The original contributions presented in the study are included in the article/Supplementary Material, further inquiries can be directed to the corresponding author.
